# Why Urban Citizens in Developing Countries Use Traditional Medicines: The Case of Suriname

**DOI:** 10.1155/2013/687197

**Published:** 2013-04-07

**Authors:** Tinde van Andel, Luísa G. Carvalheiro

**Affiliations:** ^1^Naturalis Biodiversity Center, P.O. Box 9514, 2300 RA Leiden, The Netherlands; ^2^School of Biology, University of Leeds, Leeds LS2 9JT, UK

## Abstract

The use of traditional medicines (TMs) among urban populations in developing countries and factors underlying people's decision to use TMs are poorly documented. We interviewed 270 adults in Paramaribo, Suriname, using a stratified random household sample, semistructured questionnaires, and multivariate analysis. Respondents mentioned 144 medicinal plant species, most frequently *Gossypium barbadense*, *Phyllanthus amarus*, and *Quassia amara*. 66% had used TMs in the previous year, especially people who suffered from cold, fever, hypertension, headache, uterus, and urinary tract problems. At least 22% combined herbs with prescription medicine. The strongest explanatory variables were health status, (transfer of) plant knowledge, and health status combined with plant knowledge. Other predictive variables included religion, marital status, attitude of medical personnel, religious opinion on TMs, and number of children per household. Age, gender, nationality, rural background, education, employment, income, insurance, and opinion of government or doctors had no influence. People's main motivation to use TMs was their familiarity with herbs. Given the frequent use of self-collected, home-prepared herbal medicine and the fact that illness and traditional knowledge predict plant use rather than poverty or a limited access to modern health care, the potential risks and benefits of TMs should be put prominently on the national public health agenda.

## 1. Introduction

The use of complementary and alternative medicine (CAM) has become increasingly popular in the last few decades. A 1990 survey in the United States revealed that one-third of the American adults used “unconventional therapies” [1], while natural products for health purposes were used by 19% of the population in 2002 [2]. Almost half of the Southern Australians used nonmedically prescribed alternative medicine in 2000 [3]. Reports from Western Europe suggest that 20% (The Netherlands) to 49% (France) of the population has used CAM at least once [4]. CAM types reported in these surveys included homeopathy, acupuncture, herbal medicine, dietary supplements, manual therapy, and specific prayers [2–4]. Demographic and personal factors underlying people's decision to use CAM included higher education [1, 3, 5], declining health status [5], higher income [1, 3], female, and employed [3, 6]. People's own motivations for using CAM were to prevent illness [3], curiosity, and the idea that combining it with conventional treatment would help [2]. A holistic or a spiritual health view and the belief that herbs are natural (and thus safe) also seem associated with CAM use [7]. Migrants in Europe and USA generally continue their traditional practices, including the use of traditional medicines [8–10]. Surinamese migrants in The Netherlands, interviewed in 2007, were more likely to use TMs when they believed in the existence of spirits, had been ill in the past year, and frequently visited their motherland. Their main personal motivation to use TMs was that they were part of their culture [11].

Citizens in developed countries may prefer CAM over or in combination with conventional treatment, but people in developing countries seem to have fewer options to choose from [12]. According to the World Health Organization [13], the percentage of the population in developing countries that depends on traditional medicine (TM) for their primary health care ranges from 40% (Colombia) to 90% (Ethiopia). How these figures were calculated is not revealed in the WHO statistics, nor do we know the share of herbal medicine within these TM percentages. Most figures seem to refer to rural communities that have a limited access to conventional medicine and are surrounded by environments in which herbs are easily accessible [12, 14]. However, an increasing number of people in developing countries now live in urban areas. Few of these large cities are able to provide their rapidly growing populations with the appropriate health care, which often results in increased levels of urban poverty and ill health [15]. While we are gaining insight in the use of CAM in developed countries, the use of herbal medicine or other forms of TM among urban populations in developing countries is poorly documented [12]. When 1072 citizens of Belo Horizonte (Brazil) were asked for their motivations for medicinal plant use [14], 15% said that herbs were more effective than conventional therapies, while 6% associated them with lower side effects. More than 60% of the patients from a clinic in Trujillo (Peru) said they used medicinal herbs; 35% used them more often than pharmaceuticals [16]. Of the 388 randomly selected healthy adults from Lagos (Nigeria), 67% had used herbal medicine in the past six months [17]. Researchers explained the high prevalence of herbal medicine use by massive rural-to-urban migration [14], influence of cultural and social surroundings [17], and the belief that natural products pose no risk [16, 17]. In the only study that analyzed the predictive factors for herbal medicine use in a developing country [18], 73% of the 372 randomly selected Jamaicans had used herbs in the past year. While age, employment, education, gender, health insurance, and religion predicted plant use, rural or urban residence had no influence. With the increasing popularity of CAM in developed countries and the high prevalence of TM use in developing ones, it is important for biomedical practitioners to understand their patients' motivation to use herbs or other forms of TM, the potential benefits of medicinal plants, and possible adverse effects or interactions with prescription medicine [2, 18, 19].

The current paper aims to describe use patterns and uncover the predictive variables for the use of traditional medicines among citizens of Paramaribo, Suriname. This former Dutch colony in northern South America became independent in 1975 and almost half of its inhabitants live in the capital Paramaribo (pop. 242,946 in 2004). In the period 2000–2006, 27% of the population was earning less than US$ 2 per day [20], there were 45 physicians per 100,000 people, and infant mortality was 30 per 1,000 live births [21]. Lacking sufficient means to provide for their basic needs, 59% of the urban and 63% of the rural population were considered to be poor [22]. Traditional medicines are popular in Suriname, both for physical ailments and for spiritual healing therapies. Medicinal plants as well as other forms of traditional medicine are locally known as “*oso dresi*” (home remedies). At least 400 different plant species are used, of which more than half are sold on Paramaribo's market [23, 24]. No national policies, laws, or regulations on TM exist in the country [25]. To reveal who uses home remedies in Paramaribo, what they take, and why they do this, we need to answer the following questions: (1) which types of traditional medicine are used by urban Surinamers and for what health conditions? (2) Which demographic, socioeconomic psychological factors predict the use of TMs? (3) What are people's personal motivations to use TMs? We expect that similar factors are associated with the use of TMs in Suriname as in Jamaica [18] and the use of CAM in developing countries [1–6]. Although there may be no formal policies on TM in Suriname, the opinion of politicians, religious leaders, and conventional health care practitioners may still influence people's decision regarding its use. Outcomes of this study can help to develop a national policy for TM in Suriname, but also contribute to general models that account for the use of traditional medicines among urban citizens in the developing world.

## 2. Materials and Methods

### 2.1. Sample Selection

Fieldwork was conducted in June and July 2006. Since we had no access to recent birth and death registers from Paramaribo and many inhabitants are not registered, we could not draw a random sample of all citizens. Therefore, we chose to approach participants directly in their home surroundings, using a stratified random sampling method [26, 27]. On the road map of Paramaribo [28], we selected the 94 populated quadrants and randomly placed 276 dots with a marker: three dots per quadrant in the average neighborhood, four dots in heavily populated ones (visible as many small streets), two dots in sparsely populated areas, and one dot in squares that were occupied for more than 50% by vegetation or water.

Interviews were carried out by the first author, six students, and two staff members of the Anton de Kom University in Paramaribo. Each dot on the map was visited by one or two interviewers in the late afternoon, when people generally return from work. The interviewer(s) approached the house closest to the dot on the map for a face-to-face interview with one household member of 18 years or older. When people refused to participate (mostly because of lack of time) or when there was nobody present, we selected the nearest house where someone agreed to be interviewed. Prior to the interview, participants were handed out an information sheet in Dutch that contained the aims of the study, its institutional setting, and contact information of the first author. After obtaining oral informed consent, the interview was held in Dutch, Sranantongo, Hindustani, or Javanese, according to the language preferred by the participants. All interviewees remained anonymous and received US$ 3.60 for their contribution. After exclusion of six of the 276 participants (younger than 18 years, lost or halfway terminated interviews, person was not part of the household), 270 interviews were included in our analysis. Permission to conduct this study was obtained from the Ministry of Public Health in Paramaribo (letter no. 1328).

### 2.2. Questionnaires

To find out which factors significantly influenced people's choice for traditional medicines, a pretested, semistructured questionnaire was designed  (see Supplementary Material available online at  http://dx.doi.org/10.1155/2013/687197), based on a multivariate model of human health care seeking behavior [29]. The first part contained questions related to demographic, socioeconomic, and psychosocial factors (e.g., religion, beliefs, and opinion). Participants were then asked whether they knew and used plants or other home remedies for health promotion, disease prevention, or cure. The interview continued with questions related to the occurrence of an illness the past 12 months and actions taken to overcome this ailment. Participants were asked for their opinion on the quality of both conventional health care and traditional healers in Suriname, as well as the attitude of these providers towards their patients and the standpoints of the government, church, and health care personnel towards TMs. Finally, we asked participants for their own motivation for using traditional medicines and how they obtained the raw material. The interviews were part of a larger ethnobotanical research project on Surinamese medicinal plants, carried out from January to July 2006 [23, 24]. Vernacular plant names mentioned during the interviews were connected to voucher specimens collected earlier. In a few cases, plants were collected in the respondent's garden. Duplicates of all plant vouchers were deposited at the National Herbaria of Suriname (BBS) and The Netherlands (L). Botanical research and plant export permits were obtained from the Nature Conservation Division of the Suriname Forest Service (no. 08208).

### 2.3. Statistical Analysis

To assess whether the use of traditional medicines was explained by people's social and cultural background, we tested the effect of each of the explanatory variables listed in Tables 1 and 2. The dependent variable, a dichotomous measure (yes or no), was defined as the use within the previous 12 months of any crude or processed plant or animal product for health promotion, disease prevention, or cure. As many of the variables correlated, we used generalized linear models with family binomial to examine which explanatory variables had predictive value for the use of TMs. All explanatory variables and all two-way interactions between them were evaluated in a multivariate logistic regression model selection to assess whether the use of TMs could be explained by a combination of independent variables. To select the most parsimonious model (the model that maximizes the amount of variability explained per number of variables used), we used forward and backward stepwise selection and chose the model with the lowest AIC (Akaike Information Criterion) and BIC (Bayesian Information Criterion). We then ran pairwise tests for all factorial explanatory variables that were selected in the best model. The Pearson *γ* ^2^ test was applied to examine correlations between variables. To evaluate if the occurrence of an illness affected the number of plants used for health promotion, disease prevention, or cure, we used the nonparametric Mann-Whitney *U* test after a Kolmogorov-Smirnov test indicated that the response variable was not normally distributed. In all analyses, *P* < 0.05 was considered statistically significant. Analyses were conducted using the statistical software packages IBM SPSS 19.0 and R 2.15.1 [30], using the package lme4 [31].

## 3. Results

### 3.1. Characteristics of the Response Group

The study population consisted of 89 males and 181 females, mostly born in Suriname, with a median age of 42 (Table 1). Afro-Surinamers and Hindustani formed the most prominent ethnic groups. More than half of the respondents were living with a partner; the mean number of children per household was 1.8. The majority (79%) had followed at least a few years of high school, but 44% was unemployed, although these figures include students, retired people, and housewives. Nearly three-quarters had a low income. Most were Christians (66%), followed by Hindu (14%). Only six persons listed the traditional Afro-Surinamese *winti* belief as their official religion, but more than half of the interviewees said they believed in the existence of *winti* or other spiritual beings. Almost all respondents (94%) believed that herbs had the power to heal people; less than half thought they also possessed magic power.

### 3.2. Use of Traditional Medicine

A total of 231 respondents (86%) reported to have used traditional medicines at some point in their life, while 177 (66%) had used at least one product during the past 12 months (Table 2). Only 11% stated explicitly that they did not use home remedies. More people used herbs for health promotion than for disease prevention or to cure an illness. The majority (72%) had received knowledge on herbs, mostly from their family. Books were not an important source of information; 28% of the respondents said they had never been taught about traditional medicines. People who used herbs mostly collected them in their own garden or neighborhood; 28% obtained them from family or friends, while 24% bought them on the market. Few received herbs from traditional healers. When asked for their personal motives to use home remedies, 40% said they were accustomed to use herbs; they “grew up with them” (Table 2). Other arguments were that herbs were more effective, safe and caused fewer side effects, or conventional treatment did not work. Only 12% mentioned that herbs were more accessible than prescription medicine, but just three persons said they were cheaper. Many persons gave several motives. 

### 3.3. Illnesses

Of the 174 persons who had been ill the past 12 months, 33% had a chronic disease (Table 2). Cold and related issues such as influenza and cough were the most common ailments. Of those who had been ill, 73% had used traditional medicines. Medicinal plant use was highest (70 to 100%) among people who suffered from cold, fever, hypertension, headache, uterus problems, injuries, and urinary tract problems (including sexually transmitted diseases). Those with arthritis, hernia, and skin problems less often used TM (33–35%). Most people who had been ill consulted a doctor; 44% took herbal medicine as cure. Patients generally combined several actions. One-third of the respondents knew a traditional healer; 11 persons said they were one, but only 3% had visited one in the previous year. Of those who had been sick, 22% combined herbs with prescription medicine. Another 24 patients took herbs and went to see a doctor, but did not mention the use of synthetic drugs. Nine persons visited a doctor and a traditional healer for the same illness, but neither referred to herb or drug use. Although not explicitly mentioned, persons from the latter two groups probably also used herbs in conjunction with prescription medicine. Of the people who had not been ill, 48% used medicinal plants to promote their health, 22% to prevent disease, and 6% to cure an ailment they did not report.

### 3.4. Plant Species Known and Used

All medicinal products are listed with their local and scientific names, parts used, and citation scores in the supplementary Appendix 2. At least 144 plant species were mentioned during the interviews, belonging to 66 families, with the Solanaceae as most diverse one (11 species), followed by Fabaceae (8 spp.), Asteraceae (7), and Euphorbiaceae (7). Eighteen herbal products could not be connected to a scientific name: two were native plants with undocumented vernacular names; 11 were mixtures of two or more species. Of the 144 plant species, 118 were used in the past 12 months. At least 18 plant species, several Asian mixtures, and all chemical substances were imported. Ten people said they had used herbal medicine but did not know the name of the particular plant. Bitter tonics (used by 17 respondents) consisted of a mixture of several bitter-tasting plants listed separately in Appendix 2. Apart from honey (cited 10 times), animal products like deer horn or snake fat were mentioned only sporadically. A few synthetic substances (e.g., camphor, magnesium sulfate, and Reckitt blue laundry whitener) were also considered as traditional medicines by our respondents, but they were mostly added to plant mixtures.

A red cotton cultivar (*Gossypium barbadense*) was by far the best known and most frequently used plant (Table 3). A tea from its leaves was drunk to regulate menstruation and treat uterus problems. *Phyllanthus amarus* and *Quassia amara* were two common ingredients of bitter tonics, drunk to promote one's health, treat menstruation problems, and ease the symptoms of diabetes. Since people did not always know the ingredients of their ready-made bitter tonics, these species were probably more frequently used than those listed here. Surinamese herbal medicine is not always that traditional: the Pacific fruit *Morinda citrifolia* was only promoted since the 1990s as a medicinal product, but now figures in the top five of most cited species. Processed herbal medicine imported from The Netherlands (e.g., echinaforce) was also mentioned as a “home remedy.” While 4% of the respondents did not know any medicinal plant, 61% provided information on more than three species (Table 2). People who had been ill in the previous year used significantly more plant species for disease prevention and cure than those who did not report an illness (Table 4), but the number of species used for health promotion did not differ between the two groups. More than half of our respondents used plants for health promotion, regardless whether they had been ill or not (Table 2), while ca. 35% of the respondents used plants for disease prevention and/or cure. More plants were used to cure an illness (72% of all species) and for health promotion (61%) than for disease prevention (48%), but many of the most popular species were used in all three categories. One-fifth of the respondents occasionally sent herbal medicine to friends or relatives in The Netherlands; 4% did this on a regular basis.

### 3.5. Acceptance of Herbal Medicine by Local Institutions

Our data suggest that TM is fairly well accepted by the Surinamese medical staff: 31% of our respondents said their doctor accepted both traditional healers and herbal medicine; 21 persons reported that some physicians suggested their patients to visit a traditional healer or use herbs (Table 2). Another 21% said that the opinion on TM varied among doctors: some accepted herbs but rejected traditional healers. According to 27%, their health care providers rejected both herbs and healers. Although 62% of the respondents were positive about the quality of conventional health care in Suriname, more than half were critical about the attitude of the medicinal personnel towards patients: 18 people said that they were only nice to rich people; 13 had experienced hospital staff that cursed at their patients. A 56-year-old female teacher reported: “they treat you like a dog, only if you have money and they know what kind of insurance you have” (they are willing to help you).

Our respondents generally found that traditional healers treated their patients with more respect than conventional health care providers. A 54-year-old housewife explained: “traditional healers have to treat their patients respectfully in order to get their money.”

Our results reflect the lack of an official policy on TM in Suriname: 43% of the respondents were not aware of any government opinion on the use of herbs. Several people thought that TM was accepted since politicians themselves went to traditional healers or practiced *winti* rituals, while 11% thought that both herbs and traditional healers were prohibited by law. The largest group of respondents (35%) felt that their religion accepted both herbs and healers; 30% said that this depended on the type of healers (*winti* priests were mostly not accepted) and the type of herbal medicine (ritual uses were often rejected). Just 13% said their religion opposed both herbs and healers. A 54-year-old Afro-Surinamese man, who used six different plant species to cure his chronic joint pains, told us that although his church rejected herbal medicine, his culture allowed him to use it. 

### 3.6. Statistical Analysis

Many of the explanatory variables were correlated. For example, ethnicity was correlated with religion (*γ* ^2^ = 200.9, *P* < 0.01), the belief in *winti* (*γ* ^2^ = 16.3, *P* < 0.05), knowing a traditional healer (*γ* ^2^ = 7.9, *P* < 0.05), and being taught on traditional medicines (*γ* ^2^ = 23.3, *P* < 0.01). Independent factors that explained the variability of the data in the multivariate analysis were listed in two models: a more conservative model based on BIC and a less conservative one based on AIC (Table 5). The strongest predictors for TM use were retained in the most conservative model: the occurrence on an illness, the number of medicinal plant species known, and having received information on traditional medicine.

**Figure 1:**
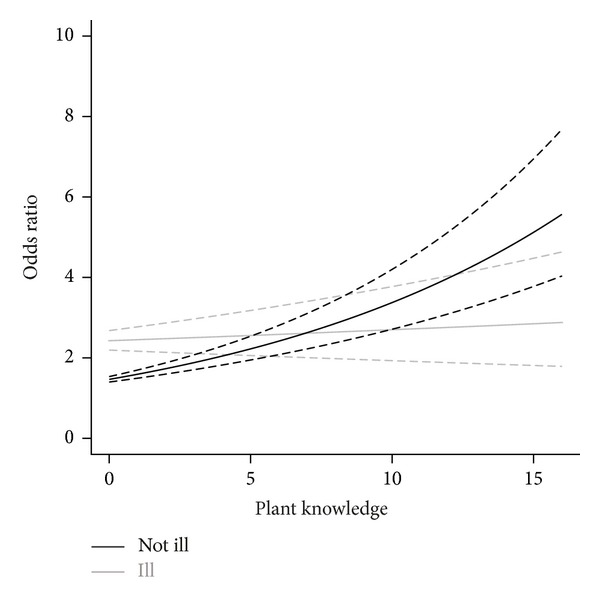
Relation between plant knowledge (no. of species used) and the use of traditional medicines (odds ratio) among people who did and did not suffer from an illness during the past 12 months. Odds ratio values were obtained from the most parsimonious model (lowest AIC and BIC), as the exponential of the estimates provided by generalized linear models with family binomial.

We also found interaction between illness and plant knowledge. People who had been ill in the past 12 months used herbs more often than those who had not; independently of whether the disease was chronic or acute. Persons who had not suffered from any illness were more likely to use herbs (for health promotion and disease prevention), particularly when their plant knowledge was higher. For those who had been ill, this interaction was not significant (Figure 1). Other variables that influenced the use of TMs were selected in the least conservative model (AIC model, Table 5). The results of this model suggest that, apart from the variables mentioned earlier, the use of herbs was linked to being Christian, single, positive about the attitude of medical staff towards patients, living in a household with many children, and feeling that one's religion accepted both herbs and traditional healers. Respondents who listed *winti* as their official religion all used herbal medicine, but this group was too small to make differences significant. Age, gender, nationality, country of birth, the number of years spent in rural areas, education, employment, income, health insurance, and the opinion of medical staff or the government towards TM did not have a significant influence on people's decision to use traditional medicine.

## 4. Discussion

### 4.1. Self Medication with Home-Prepared Herbs

Apart from the occurrence of an illness, knowledge of the healing properties of plants and their preparation methods and the transfer of such knowledge appeared to be major factors that influenced the use of traditional medicines among urban Surinamers. Even though 34% of our study group knew a traditional healer, few people consulted one for treatment or to buy herbs. Together with the long list of plant species mentioned during the interviews, these outcomes indicate that most people practice self-medication. Popular medicinal plants like red cotton (*Gossypium barbadense*), noni (*Morinda citrifolia*), and neem (*Azadirachta indica*) were not frequently sold at the Paramaribo market in 2006 [24]. They are commonly grown in city gardens and therefore do not have much commercial value, just like the weed *Phyllanthus amarus* that can be collected for free. The bitter wood of *Quassia amara*, however, needs to be brought from the interior. Its popularity among urban Surinamers was reflected in a third place on the list of botanicals sold in greatest volume on the city's herbal market [24]. People's main motivation to use TMs was their familiarity with herbs. Remarks like “I grew up with them,” “herbal medicine is my belief,” and “if tablets don't work, I do it my own way” further confirm the outcomes of our multivariate analysis. Although it seems rather obvious that individual knowledge of plants also suggests their use, Reyes-García et al. [32] prove that these two variables do not necessarily correlate. In societies that have undergone rapid socioeconomic changes, people become more integrated into the market economy and adopt (synthetic) substitutes for plants. This creates a gap between people's ethnobotanical knowledge and their actual use of plants. Our study indicates that in Paramaribo, this is not yet the case. 

In contrast to the situation in Jamaica [18], USA and Australia [1, 3, 5, 6], age, employment, income, education, gender, and health insurance did not predict herbal medicine use in Paramaribo, so we reject our hypothesis. Our data further suggest that a clear policy on TM among politicians, religious leaders, and medical personnel has the potential to influence people's health care seeking behavior. Due to the lack of uniform guidelines and contrasting opinions and practices among policy makers in Suriname, people tend to make their own decision. Our results may indicate 66% of the urban Surinamers regularly use of traditional medicine; this does not mean that they depend on herbs for their primary health care. We did not find any indication that poverty or rural-to-urban migration was related to the use of TMs. Only three respondents mentioned that herbs were cheaper than prescription medicine. Respondents with a low income more often had an insurance that covered all their health costs than people from higher income groups (*γ* ^2^ = 33.1, *P* < 0.01), and they used herbal medicine less often, although differences were not significant (*γ* ^2^ = 4.7, *P* = 0.97). 

### 4.2. Comparison with Other Studies

The high prevalence of the use of traditional medicine for colds, headache, and intestinal problems in Paramaribo was also observed in Jamaica [18, 19] and Peru [16]. Musculoskeletal conditions and chronic pains were less often treated with herbs in Suriname, but scored high on CAM use in USA [2, 5] and herb use in Jamaica [18, 19]. Lifestyle diseases like diabetes and hypertension figured highly on our lists of self-reported ailments and most popular medicinal treatments. Diabetes patients often used bitter vegetables and tonics to ease their symptoms. Research on some of these species (e.g., *Phyllanthus amarus* and *Momordica charantia*) has confirmed their ability to lower blood glucose levels [33, 34]. The frequent use of bitter plants to prevent diabetes was also reported among urban citizens in Nigeria [17], Jamaica [18], and among Ghanaian [35] and Surinamese migrants [11] in The Netherlands. Hypertension was often treated with herbal medicine in Suriname and Jamaica [19], but not with CAM in USA, as this condition was effectively managed with pharmaceutical drugs [2].

Familiarity with herbs was also the most important reason to use TMs among Surinamese migrants in The Netherlands [11]. These findings are consistent with earlier arguments that herbal medicine is a deeply rooted cultural preference [8, 16]. The Afro-Surinamese *winti* religion, based on spirit possession, ancestor rituals, and herbal baths, still plays a key role in the mental health of Surinamers [36]. Christians in Paramaribo were more likely to believe in *winti* than other religious groups (*γ* ^2^ = 15.778, *P* = 0.015). Plants employed for spiritual purposes and cultural-bound health issues (e.g., health promotion by means of bitter tonics or regular uterus cleansing) were popular among our respondents, Surinamese migrants [11], and figured prominently on the Paramaribo market [24]. Evidence for the association between holistic or spiritual beliefs and CAM use was also found in several studies in Europe, USA and Canada [5, 7, 11], and among Rastafarians (with regards to herb use) in Jamaica [18]. Apart from being familiar with herbs, urban Surinamers also used them because they were more effective, had fewer side effects than prescription medicine, and were safe because of their natural origin. Similar arguments were brought forward by herbal medicine users in Brazil [14], Peru [16], and Jamaica [19], and CAM users in UK [7], The Netherlands [11], and USA [5].

### 4.3. Strengths and Weaknesses

Strengths of this study are its multidisciplinary approach (combining plant use and public health) and the fact that it is the first multivariate analysis of herbal medicine use among urban citizens in a developing country. Weaknesses include a possible bias towards women, unemployed, and elderly people, who are likely to spend more time at home than others. As we depended on people's willingness to participate, our sample could not be strictly random. Still, door-to-door surveys like ours have long been the standard method to obtain data on public health issues [27]. In developing countries, where few people have private landline phones and reliable birth and death registers are often unavailable, this method still seems the most practical [17, 37]. Our results are not representative of the country in general. Outside of the capital, health care facilities are much more limited and poverty rates are higher [22], so rural people rely more on TMs for their primary health care than their urban compatriots. The lack of studies that statistically analyze predictive factors for the use of TMs in developing countries makes the comparison of our results with others difficult.

### 4.4. Implications for Health Policy Makers

While CAM users in developed countries generally buy processed natural products in stores [2, 3], Surinamers mostly prepare their own herbal medicine from self-collected plants or crude material bought on the market. The existence of large medicinal plant markets in urban centers in South America [14, 36, 38], Africa [39–41], Asia [42, 43], the Caribbean [44, 45], and the Middle East [46] suggests that this might be the case in many developing countries. Apparently, even when biomedical health care becomes physically more available, this does not imply that urban citizens will make exclusive use of this system, leaving health care based on traditional plant knowledge behind [47]. Researchers have raised concern about TM/CAM use in conjunction with conventional medicine [2, 6, 18, 19]. In developing countries, these risks are probably much higher, as people generally combine synthetic medicine with home-made concentrated plant extracts instead of the much more diluted homeopathic products, multivitamins, prayers, and manual therapies used in industrialized countries. 

Our data suggest that two-thirds of the urban Surinamers regularly use TMs, a percentage comparable to Peru (60%) [16] and Nigeria (67%) [17], but somewhat lower than Jamaica (73%) [18]. At least 22% of our respondents combined herb use with prescription medicine for the same illness. This emphasizes the need for studies on the safety and efficacy of frequently used Surinamese herbs. Patients who use herbs while their doctor rejects TMs (59% in our study) probably do not share this information with him. Doctors should ask about their patient's use of herbs whenever they obtain a medical history and be alert for contraindications and signs of toxicity based on potential drug-herb interactions. Examples for some of the most popular herbs are the smooth muscle contraction activity of *Gossypium barbadense* [48] and the reproductive toxicity of *Quassia amara* [49]. Moreover, several species listed in Appendix 2 are poisonous, like *Ricinus communis*, *Spigelia anthelmia*, *Manihot esculenta* (bitter cassava root), and *Catharanthus roseus*. These plants can cause poisoning even when they are not used in combination with prescription medicine [50].

Traditional medicine typically considers the whole person and the person's cultural beliefs and values in the healing process [10, 47]. In order to improve people's health condition, it is thus essential to investigate their cultural concepts of health and illness and their health care-seeking behavior. Ethnobotanists have an important role to play in medical education to raise awareness about cultural traditions that include self-treatment with medicinal plants [51]. Future research should focus on ways to improve the communication between doctors and their patients to minimize the risks of combining herbal medicine with conventional treatment, but also to encourage practitioners to negotiate treatment that is acceptable to both clinician and patient [51]. How can herbal medicine be included in health promotion and disease prevention programs?

The outcome of our study that illness and (the transfer of) traditional knowledge are the reasons why urban citizens in Suriname use TMs, rather than poverty or a limited access to modern health care, might be more universal than previously thought. The continued use of medicinal plants in urban areas, where biomedical health care is available to most citizens, has not only been observed in cities in developing countries [14, 16–18], but also among international migrant communities in USA and Europe [8, 9, 11, 46, 51]. The general idea that traditional knowledge will disappear when people enter the market society [32] is challenged by the popularity of self-medication with medicinal plant use in large urban areas [51]. Research on the potential risks and benefits of traditional medicines should therefore be put prominently on the global public health agenda.

## 5. Conclusions

Two-thirds of the urban Surinamers had used herbal medicine in the past 12 months. The use was highest among those who suffered from cold, fever, hypertension, headache, uterus, and urinary tract problems. In stead of age, gender, nationality, rural background, education, employment, income, insurance, and doctor's or government opinions, plant use was predicted by health status, (the transfer of) plant knowledge, and health status combined with plant knowledge. Other predictive variables included religion, marital status, attitude of medical personnel, religious opinion on TMs, and number of children per household. People's main motivation to use TMs was their familiarity with herbs. Given the frequent use of self-collected, home-prepared herbal medicine and the fact that illness and traditional knowledge predict the use of TMs rather than poverty or a limited access to modern health care, the potential risks and benefits of TMs should be put prominently on the national public health agenda of Suriname. The popularity of self-medication with herbal medicine in urban areas in developing countries and among migrants in Europe and USA suggests that the predictive variables for the use of TMs presented in our study might be more universal than just Suriname.

## Supplementary Material

Appendix 2: Traditional medicine mentioned by 270 randomly selected citizens of Paramaribo, Suriname, with information on scientific names, local names (English and Sranantongo), plant parts used, and citation scores.

## Figures and Tables

**Table 1:** Demographic, socioeconomic, and psychological characteristics of the 270 respondents and their use of traditional medicines during the previous 12 months.

Variables and classes	*N*	Percentage (%)	Use of TMs in the last 12 months (%)
Sex			
Male	89	33	58 (65)
Female	181	67	119 (66)
Age (median no. of years)	42		
Children in the household (mean ± st. dev.)	1.8 ± 1.8		
Lived in interior (mean nr. years ± st. dev.)	4.71 ± 9.0		
Country of birth			
Suriname	245	91	162 (66)
The Netherlands	7	3	6 (86)
Nationality			
Surinamese	245	91	161 (66)
Dutch	14	5	11 (79)
Marital status			
Married/cohabiting	149	55	91 (61)
Single (incl. widow, divorced)	107	40	73 (68)
LAT (partner lives elsewhere)	14	5	13 (93)
Ethnic group			
Afro-Surinamese	112	41	86 (77)
Hindustani	63	23	32 (51)
Mixed	54	20	40 (74)
Javanese	20	7	6 (30)
Other (White, Chinese, and Brazilian)	21	8	13 (62)
Speaks Dutch			
Well	207	77	138 (67)
Moderately	48	18	31 (65)
Badly	15	6	8 (53)
Educational level			
Low (≤primary school)	36	13	21 (58)
Moderate (high school, Com. College)	213	79	143 (67)
High (college or university)	21	8	13 (62)
Employment level			
None	120	44	76 (63)
Part time (1–4 days/week)	33	12	23 (70)
Full time (5–7 days/week)	117	43	78 (67)
Monthly income			
Low (<$550)	192	71	123 (64)
Moderate ($550–910)	39	14	24 (62)
High (>$911)	26	10	22 (85)
Religion			
None	17	6	9 (53)
Christian-Catholic	85	32	60 (71)
Christian-Protestant	93	34	69 (74)
Hindu	39	14	20 (51)
Muslim	27	10	12 (44)
Winti	6	2	6 (100)
Belief in winti/spirits			
Yes	139	51	101 (73)
No	131	49	76 (58)
Belief healing power plants			
Yes	255	94	172 (67)
No	15	6	5 (33)
Belief magic power plants			
Yes	125	46	91 (73)
No	145	54	86 (59)
Insurance			
Total coverage	119	44	77 (65)
Partial coverage	89	33	55 (61)
No insurance	53	20	42 (76)

**Table 2:** Knowledge, opinion, and practice regarding TMs among the 270 respondents.

Variables and classes	*N* (%)	Use of TMs in the last 12 months (%)
Uses medicinal herbs (sometimes)^§,∗^	231 (86)	
Used herbs the last 12 months	177 (66)	
Used herbs for health promotion^§^	147 (54)	
Used herbs for disease prevention^§^	94 (34)	
Used herbs as cure for illness^§^	98 (36)	
Knowledge of herbs*		
None	12 (4.4)	2 (8.3)
1–3 plant species	94 (35)	48 (51)
>3 plant species	164 (61)	127 (77)
Received information on herbs		
None	76 (28)	34 (45)
From family	150 (56)	116 (77)
From friends, colleagues, neighbors	32 (12)	20 (63)
From books	3 (1)	2 (67)
Illness last 12 months		
Yes	174 (64)	127 (73)
No	96 (36)	50 (52)
Duration of illness (*n* = 174)^§^		
Acute	118 (68)	86 (73)
Chronic	58 (33)	43 (74)
Type of illness (*n* = 174)^§,∗∗^		
Cold, influenza, cough	76 (44)	54 (71)
Fever	20 (11)	14 (70)
Hypertension	17 (10)	14 (82)
Arthritis, rheuma, joint pains, hernia	17 (10)	6 (35)
Headache, migraine	15 (9)	11 (73)
Stomach/intestinal problems	16 (9)	10 (63)
Diabetes	14 (8)	9 (64)
Skin problems, wounds, rash	9 (5)	3 (33)
Menstruation, uterus problems	6 (3)	6 (100)
Mental and spiritual health	6 (3)	4 (67)
Injuries, fractures	5 (3)	4 (80)
Urinary tract problems (incl. STDs)	5 (3)	4 (80)
Other	35 (20)	24 (69)
Action patient (*n* = 174)^§,∗∗^		
Doctor	116 (81)	75 (65)
Herbal medicine	76 (44)	76 (44)
Prescription medicine	52 (30)	38 (73)
Herbs + prescription medicine	38 (22)	38 (100)
Religious activities	18 (7)	12 (67)
Traditional healer	5 (3)	5 (100)
Other (diet, rest, nothing)	27 (16)	11 (41)
Sends plants to The Netherlands		
Never	199 (74)	121 (61)
Sometimes	59 (22)	44 (75)
Regularly	12 (4)	12 (100)
Sources of herbs (*n* = 231)^§,∗∗^		
Own garden or surroundings	117 (51)	90 (77)
Family, friends	65 (28)	58 (89)
Market, shop	55 (24)	44 (80)
Interior of Suriname	30 (13)	26 (87)
Traditional healer	9 (4)	6 (67)
Motives for medicinal plant use (*n* = 213)^§,∗∗^		
Accustomed to using herbs	92 (40)	76 (83)
Plants work better than pills	71 (31)	54 (76)
Herbs are safe	51 (22)	39 (76)
Less side effects	52 (23)	42 (81)
Doctor or pills cannot cure my illness	33 (14)	17 (52)
Other (e.g., cheaper, easy access)	27 (12)	20 (74)
Knows traditional healer		
No	178 (66)	111 (62)
Yes	92 (34)	66 (72)
Quality of traditional healers		
Good	125 (46)	86 (69)
Depends on healer and illness	108 (40)	73 (68)
Bad	18 (7)	9 (50)
Attitude of traditional healer towards patients		
Good	139 (51)	92 (66)
Depends on person	66 (24)	50 (76)
Bad	2 (1)	8 (61)
Quality of conventional health care		
Good	167 (62)	108 (65)
Depends on doctor and illness	88 (33)	64 (73)
Bad	9 (3)	3 (33)
Attitude of health personnel towards patients		
Good	93 (34)	68 (73)
Depends on doctor or nurse	144 (53)	89 (62)
Bad	30 (11)	18 (60)
Opinion of doctors on traditional medicine		
Both healers and herbs accepted	83 (31)	60 (72)
None accepted	73 (27)	43 (59)
Depends on doctor	31 (11)	23 (74)
Herbs accepted, (some) healers not	27 (10)	21 (78)
Does not know doctor's opinion	56 (21)	30 (54)
Opinion of religion on traditional medicine		
Both healers and herbs accepted	95 (35)	69 (73)
Herbs accepted, (some) healers not	70 (26)	46 (66)
Does not know	66 (24)	40 (61)
None accepted	35 (13)	19 (54)
Depends on type of healer and priest	11 (4)	3 (75)
Opinion of government on traditional medicine		
Both healers and herbs accepted	108 (40)	76 (70)
Forbidden by law	31 (11)	21 (68)
Depends on healer and politician	3 (1)	3 (100)
Herbs accepted, (some) healers not	7 (3)	7 (6)
Is not aware of govt. opinion	116 (43)	70 (60)

*As most traditional medicines consist of medicinal plants, the term “herbs” is used here.

**More answers were possible so sum of percentages may be over 100%.

^§^Variable not included in the multivariate analysis.

**Table 3:** Most frequently mentioned and used medicinal plant species by our study group.

Species (voucher number)	Family	Local name (Sranantongo)	Plant part	Uses	Times cited (%)	Used (%)
*Gossypium barbadense* (TVA 4921)	Malvaceae	Redi katoen, red cotton	Leaves	Urinary tract problems, regulate menstruation, cleanse uterus, hypertension	58 (21)	37 (14)

*Phyllanthus amarus* (TVA 4870)	Phyllanthaceae	Fini bita	Entire plant	Cleanse uterus, regulate menstruation, diabetes, hypertension	48 (18)	34 (13)

*Quassia amara* (TVA 4829)	Simaroubaceae	Kwasibita	Wood	Fever, aphrodisiac, malaria, bitter tonic	48 (18)	32 (12)

*Azadirachta indica* (TVA 5539)	Meliaceae	Neem	Leaves	Hypertension, diabetes, skin problems, malaria	39 (14)	19 (7)

*Morinda citrifolia* (TVA 4761)	Rubiaceae	Noni	Fruit	Cancer, diabetes, HIV, skin problems	36 (13)	19 (7)

*Cymbopogon citratus* (TVA 4839)	Poaceae	Citroengras, lemongrass	Leaves	Cold, fever, cough, flu	35 (13)	20 (7)

*Momordica charantia* (TVA 5494)	Cucurbitaceae	Sopropo (wild)	Entire plant	Diabetes, hypertension, cleanse uterus, stomach problems	31 (11)	16 (6)

*Allium sativum *	Alliaceae	Knoflook, garlic	Bulb	Hypertension, diabetes, fever, spiritual problems	29 (11)	19 (7)

—	—	Bita (bitter tonics)	Various species	Cleanse uterus, regulate menstruation	29 (11)	17 (6)

*Peperomia pellucida* (TVA 4851)	Piperaceae	Konsaka wiwiri	Entire plant	Eye infection, fever, asthma, nausea	20 (7)	12 (4)

*Cocos nucifera *	Arecaceae	Kokosnoot, coconut	Fruitshell, coconut milk, oil	Hypertension, skin problems, hair oil, cold, diarrhea	17 (6)	10 (4)

*Eugenia uniflora* (TVA 5330)	Myrtaceae	Monkimonki kersi	Leaves	Cold, headache, sore throat, anaemia, fever	17 (6)	8 (3)

*Annona muricata* (TVA 5150)	Annonaceae	Zuurzak	Leaves	Sleeplessness, depression, anxiety, heart problems	16 (6)	9 (3)

*Scoparia dulcis* (TVA 4966)	Scrophulariaceae	Sisibi wiwiri	Entire plant	Sore throat, fever, toothache, hypertension, laxative, hepatitis	16 (6)	9 (3)

*Aloe vera *	Asphodelaceae	*Aloe vera *	Leaves	Wounds, skin infections, rash, hair problems, malaria, diabetes	13 (5)	8 (3)

*Psidium guajava* (TVA 5129)	Myrtaceae	Goyave	Fruit, leaves	Diarrhea, dysentery, fever, cold, stomach pains, cleanse uterus	13 (5)	6 (2)

*Saccharum officinarum *	Poaceae	Suikerriet, ingi tjen, melasse	Stem, leaves, syrup, juice	Cold, cough, flu, asthma, skin infection, spiritual problems	12 (4)	11 (4)

*Citrus aurantifolia* (TVA 5132)	Rutaceae	Lemmetje	Fruit	Cold, cough, flu, asthma, dysentery, skin infections	12 (4)	10 (4)

—	—	Bitter vegetables	Various species	Health promotion, anaemia, diabetes, migraine, stress	12 (4)	7 (3)

—		Herbal bath	Various species	Spiritual problems, good luck	12 (4)	7 (3)

**Table 4:** Effect of the occurrence of an illness in the previous 12 months on the number of plant species used for health promotion, disease prevention, and cure. Mean numbers of plant species (±standard error) used are provided. Data on number of plants used were not normally distributed, so *P* values were derived form a Mann-Whitney *U* test.

Health status	*N* (%)	Plants used for health promotion (mean no. spp. ± std. error)	Plants used for disease prevention (mean no. spp. ± std. error)	Plants used for cure (mean no. spp. ± std. error)
Ill last 12 months	174 (64)	1.10 ± 1.3	0.71 ± 1.1	1.05 ± 1.3
Not ill	96 (36)	0.81 ± 1.2	0.47 ± 1.2	0.12 ± 0.5
Total	**270 (100)**	**1.0 ± 1.3**	**0.62 ± 1.2**	**0.72 ± 1.1**
*P* value	—	0.44	0.006	<0.0001

**Table 5:** Variables that best explained the variability in use of traditional medicines in the last 12 months. Information on the most parsimonious model obtained when using AIC or BIC during model selection is presented. Negative values for the deviance to the reference level imply a lower probability of TM use than the reference group (deviance = 0).

Variables	*n* (%)^§^	Slope of covariates	Deviance to reference	Std. error	Pairwise comparison with reference level		
					*T* value	*P* value	
Best model based on AIC							

Religion							
Christian (reference level)	178 (66)		0	—	—	—	
Winti	6 (2)		−0.1676	0.1883	−0.9	0.3742	
Hindu	39 (14)		−0.2734	0.0776	−3.5	0.0005	***
Muslim and other	47 (17)		−0.2180	0.0704	−3.1	0.0022	**
Number of children in household^§§^		0.0273		0.0140	20.	0.0522	.
Plant knowledge (number of spp.)^§§^		0.0108		0.0160	0.7	0.5020	
Illness last 12 months							
Yes (reference level)	174 (64)		0	—	—	—	
None	96 (36)		−0.5032	0.1101	−4.6	7.53*E* − 06	***
Marital status							
Single or LAT (reference level)			0	—	—	—	
Married/cohabiting			−0.1173	0.0530	−2.2	0.0279	*
Received information on herbs							
From family, friends, and so on (reference level)	194 (72)		0	—	—	—	
None	76 (28)		−0.1946	0.0591	−3.3	0.0011	**
Attitude medical staff towards patients							
Good (reference level)	94 (35)		0	—	—	—	
Does not know	3 (1)		0.0531	0.2466	0.2	0.8298	
Has experienced bad attitude	173 (64)		−0.1355	0.0550	−2.5	0.0143	*
Opinion of religion on traditional medicine							
Positive (reference level)	95 (35)		0	—	—	—	
Critical (depends on priest/type of TM)	74 (27)		−0.1994	0.0691	−2.9	0.0042	**
Does not know	66 (24)		−0.1590	0.0715	−2.2	0.0271	*
Negative	35 (13)		−0.2110	0.0852	−2.5	0.0139	**
Illness ∗ plant knowledge							
Yes (reference level)	174 (64)		0	—	—	—	
No	96 (36)		0.0727	0.0235	3.1	0.0022	**

Best model based on BIC							

Received information on herbs							
From family, friends, and so on	194 (72)		0				
None	76 (28)		−1.0551	0.3135	−3.4	0.0008	***
Illness last 12 months							
Yes	174 (64)		0				
None	96 (36)		−2.7597	0.7834	−3.5	0.0004	***
Plant knowledge (number of spp.)^§§^		0.1102		0.0941	1.2	0.2415	
Illness last 12 months ∗ plant knowledge							
Yes	174 (64)		0				
None	96 (36)		0.4598	0.1870	2.5	0.0139	*

^§^Because of rounding, percentages do not always total 100.

^§§^Continuous variable.

*Level of significance:  .: almost significant; *P < 0.05; **P < 0.01; ***P < 0.001.

*P* values < 0.05 are considered significant.
