# Supplementary material for: Why Urban Citizens in Developing Countries Use Traditional Medicines: The Case of Suriname
Source: Evid Based Complement Alternat Med. 2013 Apr 7;2013:687197. doi: 10.1155/2013/687197 (PMC3638607; doi:10.1155/2013/687197)
Supplement: Supplementary file 1 — Appendix 2: Traditional medicine mentioned by 270 randomly selected citizens of Paramaribo, Suriname, with information on scientific names, local names (English and Sranantongo), plant parts used, and citation scores. [file 687197.f1.pdf]

Appendix 2. Traditional medicines mentioned during the 270 interviews in Paramaribo, Suriname (2006).

MEDICINAL PLANTS

Local name (s)	English name	Plant part	Species	Family	Mentioned	Used in the past 12 months
redi katoen	red cotton	leaves	*Gossypium barbadense* L.	Malvaceae	58	37
fini bita	carry me seed	entire plant	*Phyllanthus amarus* Schumach. & Thonn.	Phyllanthaceae	48	34
kwasibita	bitterwood, bitter ash	wood	*Quassia amara* L.	Simaroubaceae	48	32
neem	neem	leaves	*Azadirachta indica* A.Juss.	Meliaceae	39	19
noni	noni	fruit	*Morinda citrifolia* L.	Rubiaceae	36	19
citroengras	lemongrass	leaves	*Cymbopogon citratus* (DC.) Stapf	Poaceae	35	20
sopropo, wilde	bitter melon (wild form)	entire plant	*Momordica charantia* L.	Cucurbitaceae	31	16
knoflook	garclic	bulb (imported)	*Allium sativum* L.	Alliaceae	29	19
konsaka wiwiri	pepper elder	entire plant	*Peperomia pellucida* (L.) Kunth	Piperaceae	20	12
kokosnoot	coconut	fruit, shell, juice, oil from seed	*Cocos nucifera* L.	Arecaceae	17	10
monkimonki kersie	Surinam cherry	leaves	*Eugenia uniflora* L.	Myrtaceae	17	8
zuurzak	soursop	leaves	*Annona muricata* L.	Annonaceae	16	9
sisibi wiwiri	sweet broom	entire plant	*Scoparia dulcis* L.	Scrophulariaceae	16	9
aloe vera	aloes	leaves	*Aloe vera* (L.) Burm.f.	Asphodelaceae	13	8
guyave	guava	fruit, leaves	*Psidium guajava* L.	Myrtaceae	13	6
suikerriet, ingi tjen, melasse	sugarcane	stem, leaves, syrup, juice	*Saccharum officinarum* L.	Poaceae	12	11
lemmetje	lime	fruit	*Citrus aurantiifolia* (Christm.) Swingle	Rutaceae	12	10
swa alanya	bitter orange	fruit	*Citrus aurantium* L.	Rutaceae	11	8
jarakopi, koorsuwiwiri	-	leaves	*Siparuna guianensis* Aubl.	Siparunaceae	11	8
soepgroente	celery	leaves	*Apium graveolens* L.	Apiaceae	10	10
blaka uma	black sage	leaves	*Cordia curassavica* (Jacq.) Roem. & Schult.	Boraginaceae	10	7
gember (stroop)	ginger	rhizome (syrup)	*Zingiber officinale* Roscoe	Zingiberaceae	10	7
slabriki	candle bush	flowers, leaves	*Senna alata* (L.) Roxb.	Fabaceae	10	4
sinaas	orange	fruit, fruit shell, leaves	*Citrus sinensis* (L.) Osbeck	Rutaceae	9	7
anijsblad	-	leaves	*Piper* spp.	Piperaceae	9	6
papaya	pawpaw	fruit (green and ripe)	*Carica papaya* L.	Caricaceae	9	5
bospapaya, congo pompu	pumpwood	leaves	*Cecropia sciadophylla* Mart.	Cecropiaceae	9	2
kumis kutching	cat's whiskers	leaves	*Orthosiphon aristatus* (Blume) Miq.	Lamiaceae	8	5
kasjoe	cashew	fruit, bark	*Anacardium occidentale* L.	Anacardiaceae	8	4
nootmuskaat	nutmeg	seed (imported)	*Myristica fragrans* Houtt.	Myristicaceae	7	6
bita wiri	bitterleaf	leaves	*Cestrum latifolium* Lam.	Solanaceae	7	5
banaan	banana	fruit (green and ripe)	*Musa* sp.	Musaceae	7	5
loango tete	-	wood	*Aristolochia consimilis* Mast.	Aristolochiaceae	7	3
krappa	crabwood	bark, oil from seed	*Carapa guianensis* Aubl.	Meliaceae	7	3

grapefruit	grapefruit	fruit	*Citrus paradisi* Macfad.	Rutaceae	6	5
batoto bita	wild tomato	entire plant	*Physalis angulat* a L.	Solanaceae	6	4
antroewa	African eggplant	fruit	*Solanum macrocarpon* L.	Solanaceae	6	4
brudu wiri	St. John's Bush	leaves	*Justicia secunda* Vahl	Acanthaceae	6	3
pomme cithere	Jewish plum	fruit (green and ripe)	*Spondias dulcis* Parkinson	Anacardiaceae	5	5
parabita	-	leaves	*Solanum leucocarpon* Dunal	Solanaceae	5	4
citroen	lemon	fruit	*Citrus limon* (L.) Burm. f.	Rutaceae	5	3
senneblad	senna leaves	leaves (imported)	*Senna* cf. *alexandrina* Mill.	Fabaceae	5	3
pampoen	pumpkin	flowers, leaves	*Cucurbita moschata* Duchesne	Cucurbitaceae	5	2
kruidnagel	cloves	flowers	*Syzygium aromaticum* (L.) Merr. & L.M.Perry	Myrtaceae	5	2
hoepelolie	-	bark, oil from bark	*Copaifera guyanensis* Desf.	Fabaceae	4	4
birambi, fransman / lange	bilimbi	leaves, fruit	*Averrhoa bilimbi* L.	Oxalidaceae	4	3
mintwater, mintolie	mint oil	oil from leaves (imporrted)	*Mentha* sp.	Lamiaceae	4	3
tulsi	holy tulsi	leaves	*Ocimum tenuiflorum* L.	Lamiaceae	4	3
jamoen	jamoon	fruit, bark	*Syzygium cumini* (L.) Skeels	Myrtaceae	4	3
sangrafu	spiral ginger	stem	*Costus arabicus* L. and *C. scaber* Ruiz & Pa	Costaceae	4	2
loksi	West Indian locust	bark	*Hymenaea courbaril* L.	Fabaceae	4	2
castorolie, wonderolie	castor oil	oil from seed	*Ricinus communis* L.	Euphorbiaceae	4	2
wit ede	American burnweed	entire plant	*Erechtites hieracifolia* (L.) Raf.	Asteraceae	4	1
eiwitblad	flat-on-the-ground	entire plant	*Microtea debilis* Sw.	Phytolacaceae	4	1
tamarindeblad	tamarind	leaves	*Tamarindus indica* L.	Fabaceae	4	1
kotomisie (witte)	Madagascar periwinkle	entire plant (white-flowered form)	*Catharantus roseus* (L.) G.Don	Apocynaceae	4	0
rode croton, tufesi wiwiri	copperleaf	leaves	*Acalypha wilkesiana* Müll.Arg.	Euphorbiaceae	3	3
goal para thee	green tea	leaves (imported)	*Camellia sinensis* (L.) Kuntze	Theaceae	3	3
tajerblad	eddoe, arrowleaf elephant ear	leaves	*Colocasia esculenta* (L.) Schott and *Xantho*	Araceae	3	3
steranijs	star anise	seeds (imported)	*Illicium verum* Hook. f.	Schisandraceae	3	3
dagublad	water spinach	entire plant	*Ipomoea aquatica* Forssk.	Convolvulaceae	3	3
bittere cassave	bitter cassava	tuber	*Manihot esculenta* Crantz.	Euphorbiaceae	3	3
sopropo, gecultiveerde	bitter melon (cultivated form)	fruit	*Momordica charantia* L.	Cucurbitaceae	3	3
uien	onion	bulb (imported)	*Allium cepa* L.	Alliaceae	3	2
sekrepatu wiwiri	water hemp	entire plant	*Ayapana triplinervis* (Vahl) R.M.King & H.Ro	Asteraceae	3	2
wonderblad	leaf-of-life	leaves	*Bryophyllum pinnatum* (Lam.) Oken	Crassulaceae	3	2
kalebas	calabash	fruit	*Crescentia cujete* L.	Bignoniaceae	3	2
kwintu, sneki wiwiri	Mexican coriander	entire plant	*Eryngium foetidum* L.	Apiaceae	3	2
sukrutante	-	leaves	*Tilesia baccata* (L.) Pruski	Asteraceae	3	2
krerekrereblad, rode	peacock flower	leaves (red-flowered form)	*Caesalpinia pulcherrima* (L.) Sw.	Fabaceae	3	1
komkommer, gecultiveerde	cucumber	fruit	*Cucumis sativus* L.	Cucurbitaceae	3	1
rosa wiri	false daisy	entire plant	*Eclipta prostrata* (L.) L.	Asteraceae	3	1

west-indische kers	West Indian cherry	fruit	*Malpighia glabra* L.	Malpighiaceae	3	1
manja	mango	leaves, bark	*Mangifera indica* L.	Anacardiaceae	3	1
anijszaad	anise seed	seed (imported)	*Pimpinella anisum* L.	Apiaceae	3	1
pedrekoe	-	fruit	*Xylopia discreta* (L.f.) Sprague & Hutch.	Annonaceae	3	0
kattestaart	chenille plant	leaves	*Acalypha hispida* Burm. f.	Euphorbiaceae	2	2
komijn	cumin	seed (imported)	*Cuminum cyminum* L.	Apiaceae	2	2
Echinaforce Dr. Vogel	Echinaforce	processed extract (imported)	*Echinacea* spp.	Asteraceae	2	2
asafoetida	asafoetida	root paste (imported from India)	*Ferula assa-foetida* L.	Apiaceae	2	2
napi	sweet potato	tuber	*Ipomoea batatas* (L.) Poir.	Convolvulaceae	2	2
tomaat	tomato	fruit	*Lycopersicum esculentum* Mill.	Solanaceae	2	2
agoma wiwiri	American nightshade	entire plant	*Solanum americanum* Mill.	Solanaceae	2	2
kastanje blad	breadnut	leaves	*Artocarpus altilis* (Parkinson ex F.A.Zorn) F(	Moraceae	2	1
kowru ati	-	entire plant	*Begonia glabra* Aubl.	Begoniaceae	2	1
peper	red pepper	fruit juice	*Capsicum anuum* L.	Solanaceae	2	1
feyfi finga	bread and cheese	leaves	*Paullinia pinnata* L.	Sapindaceae	2	1
avocado	avocado pear	leaves	*Persea americana* Mill.	Lauraceae	2	1
rozebottelthee	rose-hip	fruit (imported)	*Rosa canina* L.	Rosaceae	2	1
duruduru	-	wood	*Tanaecium bilabiatum* (Sprague) L.G.Lohma	Bignoniaceae	2	1
merkiwiwiri	chickenweed	entire plant	*Euphorbia thymifolia* L.	Euphorbiaceae	2	0
nengre kondre pepre	grains of paradise	seed	*Aframomum melegueta* K.Schum.	Zingiberaceae	1	1
prei	leek	leaves	*Allium ampeloprasum* L.	Amaryllidaceae	1	1
spinazie	amaranth	leaves	*Amaranthus blitum* L.	Amaranthaceae	1	1
ananas	pineapple	fruit	*Ananas comosus* (L.) Merr.	Bromeliaceae	1	1
birambi ster	star fruit	fruit	*Averrhoa carambola* L.	Oxalidaceae	1	1
kaneel	cinnamon	bark	*Cinnamomum verum* J. Presl.	Lauraceae	1	1
Chinese tayer	eddoe	tuber	*Colocasia esculenta* (L.) Schott	Araceae	1	1
ingi pipa	-	fruit	*Couratari* sp.	Lecythidaceae	1	1
hrdi, geelwortel	turmeric	rhizome	*Curcuma longa* L.	Zingiberaceae	1	1
laos	laos	root	*Alpinia galanga* (L.) Wildd.	Zingiberaceae	1	1
cactus	creeping spurge	entire plant	*Euphorbia neriifolia* L.	Euphorbiaceae	1	1
sojabonen	soy beans	seeds (imported)	*Glycine max* (L.) Merr.	Fabaceae	1	1
kakanoto	physic nut	seed	*Jatropha curcas* L.	Euphorbiaceae	1	1
brantimakka	manatee bush	leaves	*Machaerium lunatum* (L.f.) Ducke	Fabaceae	1	1
komkommer (wild)	wild cucumber	fruit	*Melothria pendula* L.	Cucurbitaceae	1	1
ginseng	ginseng root	root (imported)	*Panax ginseng* C.A. Mey	Araliaceae	1	1
foengoe	-	hair from leaves	*Parinari campestris* Aubl.	Chrysobalanaceae	1	1
bakru wiri	Guinea hen weed	entire plant	*Petiveria alliacea* L.	Phytolacaceae	1	1
paanblad	betle leaf	leaves	*Piper betle* L.	Piperaceae	1	1

zwarte peper	black pepper	seeds (imported)	*Piper nigrum* L.	Piperaceae	1	1
fukufukuminti	Mexican mint	leaves	*Plectranthus amboinicus* (Lour.) Spreng.	Lamiaceae	1	1
bakru wiri	clammy bur	entire plant	*Priva lappulacea* (L.) Pers.	Verbenaceae	1	1
ramenas	black radish	tuber	*Raphanus raphanistrum* ssp. *sativus* (L.) D(	Brassicaceae	1	1
roosolie, rozenblad	rose oil	flowers, in oil (imported)	*Rosa* sp.	Rosaceae	1	1
vlier	American elderberry	leaves	*Sambucus canadensis* L.	Adoxaceae	1	1
bostomaat	-	fruit	*Solanum stramoniifolium* Jacq.	Solanaceae	1	1
drunguman	worm bush, pink root	entire plant	*Spigelia anthelmia* L.	Loganiaceae	1	1
kamferbita	camphor bitters	entire plant	*Unxia camphorata* L. f.	Asteraceae	1	1
blakaston	Chinese jujube	fruit (imported)	*Ziziphus jujuba* Mill.	Rhamnaceae	1	1
gotu kola	centella	entire plant (imported)	*Centella asiatica* (L.) Urb.	Apiaceae	1	1
bamboe	bamboo	leaves	*Bambusa vulgaris* Schrad.	Poaceae	1	0
kusuwe	anatto	leaves, fruit	*Bixa orellana* L.	Bixaceae	1	0
watermeloen	watermelon	fruit	*Citrullus lanatus* (Thunb.) Matsum. & Nakai	Cucurbitaceae	1	0
mauby	mauby	bark (imported from Trinidad)	*Colubrina arborescens* (Mill.) Sarg., C. ellipt	Rhamnaceae	1	0
tafrabon	table tree	leaves	*Cordia tetrandra* Aubl.	Boraginaceae	1	0
podosiri	asai fruits	fruit juice	*Euterpe oleraceae* Mart.	Arecaceae	1	0
ginkgo biloba	ginkgo	leaves (imported from China)	*Ginkgo biloba* L.	Ginkgoaceae.	1	0
balsemien	balsamine	entire plant	*Impatiens* sp.	Balsaminaceae	1	0
faya lobi	flame of the woods	leaves, flowers	*Ixora coccinea* L.	Rubiaceae	1	0
brandnetel	stinging nettle	leaves	*Laportea aestuans* (L.) Chew	Urticaceae	1	0
blaka tiki menti	bushy matgrass	leaves	*Lippia alba* (Mill.) N.E. Brown	Verbenaceae	1	0
brokobaka	mile-a-minute	leaves	*Mikania micrantha* Kunth	Asteraceae	1	0
mokomoko	-	leaves	*Montrichardia arborescens* (L.) Schott	Araceae	1	0
lotuszaad	lotus	seed	*Nelumbo nucifera* Gaertn.	Nelumbonaceae	1	0
tabak	tobacco	leaves	*Nicotiana tabacum* L.	Solanaceae	1	0
smeri wiwiri	wild sweet basil	entire plant	*Ocimum campechianum* Mill.	Lamiaceae	1	0
tiensensi wiri	creeping peperomia	entire plant	*Peperomia rotundifolia* (L.) Kunth	Piperaceae	1	0
fowru doti	bird vine	entire plant	*Phthirusa stelis* (L.) Kuijt	Loranthaceae	1	0
granaatappelschil	pomegranata	fruit shell	*Punica granatum* L.	Lythraceae	1	0
boulanger, aubergine	eggplant	fruit	*Solanum melongena* L.	Solanaceae	1	0
aardappel	potato	tuber (imported)	*Solanum tuberosum* L.	Solanaceae	1	0
amandel	almond	leaves	*Terminalia catappa* L.	Combretaceae	1	0
malva	sleepy morning	entire plant	*Waltheria indica* L.	Malvaceae	1	0
mais	maize	seed, leaves, flower	*Zea mays* L. subsp. *mays*	Poaceae	1	0

UNKNOWN PLANT SPECIES AND MIXTURES

bita	bitters	various species	mixture for health promotion, uterus cleansing and aphrodisiac	29	17
unknown	-	unknown plant species		18	10
bittere groenten	bitter vegetables	various species of leaves	single species, boiled as vegetable	12	7
kruidenbad	herbal bath	various leaves	mixture	12	7
kowru dresie	laxative	plants and (imported) chemicals	mixture (strong laxative)	6	4
uma wasi	genital steam bath	various species of leaves and/or ba	mixture (genital steam bath)	6	4
Chinese kruiden	Chinese herbs	various Chinese species	imported by Chinese shops	4	3
djumu, djamu	jamu	various Javanese rhizomes	imported by Javanese and home-made	4	2
fruit	fruits (general)	various fruit species		3	2
chinees poeder kruiden	Chinese 5-spice powder	spice mixture	mixture of black pepper, cloves, star anise, cinnamon and fennel.	2	2
massala	massala	various Asian spices	spice mixture, imported from India or home-made	2	1
kwakoe kiri alende	-	unknown plant species	bark against hepatitis	2	0
odany jewa	-	several plant species	commercially processed herbal medicine	1	1
kerrie	curry powder	various Asian rhizomes	spice powder (curry), imported from India or home-made	1	1
man batra	aphrodisiac mixture	various wood, barks, roots	aphrodisiac mixture soaked in alcohol	1	1
murudresie	uterus cleansing mixture	various species	mixture of native species for womb cleansing	1	0
kargasok	kargasok	yeast product	imported homeopathic product	1	0
ANIMAL PRODUCTS					
honing	-	honey	imported or wild-collected	10	10
diatoe	-	deer horn	probably *Odocoileus virginianus* Zimmermann 1780	3	3
aboma vet	-	fat from anaconda snake	*Eunectes murinus* L. (1758)	1	0
CHEMICAL OR OTHER PRODUCTS					
kamfer	-	synthetic camphor	imported	2	2
blauwsel	-	Reckitt blue synthetic dye	imported	1	1
dondersteen	-	stone axe	found in prehistoric Amerindian sites	1	1
bere te	-	unknown substance	umbilical cord? (literal translation of Sranantongo)	1	1
epsomzout	-	magnesium sulfate	imported	1	1
pemba	-	white clay (kaolin)	harvested in savanna belt	1	1
petroleum	-	kerosine	imported	1	0
